# Pelvic Cellulitis*Read before the Buffalo Obstetrical Society, May 24, 1887.

**Published:** 1887-09

**Authors:** Walter D. Greene


THE BUFFALO MEDICAL AND SURGICAL JOURNAL

VOL. XXVIII. SEPTEMBER, 1887. No. 2.

### Original Communications.

### *PELVIC CELLULITIS.**

By WALTER D. GREENE, M. D.

Nearly every woman who has borne children, or has experienced the parturient process in any of its stages, has suffered, more or less, from pelvic cellulitis, and from the fact of its frequency in its various degrees of severity, makes it especially of interest to the obstetrician and gynecologist. Its history is somewhat peculiar, having been discovered and forgotten three times in past centuries, until the researches of Churchill and others, in 1843 and '44, again brought it to light. Pelvic cellulitis consists of an inflammation of the loose cellular tissue surrounding the uterus and extending between the layers of serous membrane, constituting the broad ligaments. As this tissue is very plentiful in the cavity of the pelvis, when the seeds of inflammation are sown, they quickly spread and usually involve the whole uterine cavity. This tissue exists everywhere in the pelvis around the pelvic organs, except between the peritoneum and uterus, where so little, if any, exists, that its presence has been denied by some observers. Different writers have given the disease the following names: parametritis, periuterine, phlegmon-inflammation of broad ligaments, pelvic abscess and pelvic cellulitis, the

* Read before the Buffalo Obstetrical Society, May 24, 1887.

latter by Sir James Simpson. The majority of the British medical men considered the latter name as applicable to any inflammation of cellular tissue within the pelvis, which would include psoas and iliac abscesses. Because of the indefiniteness, Dr. Thomas prefers periuterine rather than pelvic cellulitis. We are to understand, therefore, by these several appellations an inflammation of the adipose and areolar tissues, connecting the peritoneum with the floor of the pelvis, or the rectum bladder and uterus. Although every case of pelvic cellulitis is not traumatic in origin, it is nevertheless a fact that many of them are based on an infection from traumatic causes. In laceration of the cervix during labor the wound is liable to extend to the cellular tissue. How often do we find cicatricial tissue as a result of cellulitis, and the os uteri drawn around sometimes nearly to a right angle to the vaginal canal? Also minor operations upon or near cervix, and the treatment of the os with tents are liable to give rise to this disease. Pessaries form another prolific cause, and especially the stem variety. When they are ill-fitting and are left in situ for any length of time, cellulitis is liable to ensue with general peritonitis as well. The introduction of a sound up to the fundus of the uterus and endeavors to restore displacements of that organ are to be numbered with the causes, but according to statistics parturition at term and abortion cause one-half to two-thirds of the cases. It is certainly comparatively rare, except after the parturient process; where a predisposition exists, cold, fatigue, overwork, menstruation, etc., will develop it as immediate and exciting causes only. That it is not an idiopathic affection seems to be pretty generally conceded, although in nullipara, young and old, suppurating pelvic exudations of obscure origin are occasionally seen. When abscesses do occur, they are more often to be found in the broad ligaments than elsewhere—including ovaries and Fallopian tubes. In post mortem examinations these parts are often found in a state of suppuration, the tubes filled with pus or sero-purulent material. In a report of fourteen post mortem cases, in twelve the abscesses were found in the broad ligaments. Uncomplicated pelvic cellulitis generally runs a course of from two to four weeks, and ends in resolution or suppuration, only more often in the former. Should it become complicated with peritonitis, endometritis, ovaritis, etc., it may become chronic, or suppurate and discharge pus for years after having dissected its way out by long sinuous routes. The points of exit of the pus may be through the abdominal walls, the bladder, rectum, vagina, urethra or uterus, the floor of the pelvis, pelvic foramina, sacro-sciatic notch, or through to pelvic roof into the peritoneal cavity. It comparatively seldom opens directly into the vagina, but burrows beside that canal and reaches the labia majora. I have seen one case of this kind into which I passed a sound four and one-half inches. In thirty-seven cases treated by Dr. McClintock, which suppurated, twenty discharged in the iliac region, two above the pubes, one in vaginal region, one beside the anus, six per vagina, five per anus, and two into the bladder. In acute cases, and especially those following parturition, the most prominent symptoms are first a chill followed by fever, the thermometer under the tongue frequently registering 104°, with fast pulse, pain, although not usually severe, difficult and painful micturition and often slight discharge of blood from the uterus. Vaginal examination at this time will reveal a hot surface with a tendency to dryness, with absence of tumor or hardness. As the disease advances, however, infiltration can be discovered and generally laterally to the uterus. More or less circumscribed tumors form, and as time goes on they gradually merge into each other, until the uterus seems imbedded in a hard tumor and becomes fixed. Often the limb on the side most affected is drawn up, but this is not a constant symptom. The bowels are generally constipated and vomiting is often present; headache, very rare. The symptoms of pelvic cellulitis of long standing are sometimes exceedingly slight, large tumors surrounding the uterus in women who do not consider themselves seriously sick. These are cases which develop very slowly without fever and very little pain; who have dragging pains in the thighs and back, consequent upon the pressing of the tumor upon the pelvic nerves. These are patients who say they simply endure life, but do not enjoy it. They are troubled with difficult defecation, and have pain during coition; capricious appetite and shortness of breath, constipation and sleeplessness. Almost all of these cases refer the origin of their trouble to parturition, and they are generally correct. During the parturient act possibly some slight tear of the os or upper vagina may have occurred, which developed a slow traumatic cellulitis, although it must be confessed that most cases developing cellulitis after parturition are of an acute instead of a chronic nature. Those women who are sterile generally have cellulitis in a chronic form, and usually remain so even after restoration to health, if the cellulitis has been of long standing. On the other hand, when the disease is of an acute nature and ends in either resolution or abscess in two or three weeks, the power of conception is not lost. In chronic forms, the woman suffers for years, until some slight cold during menstruation or overexertion, or other cause lights up the disease anew, an abscess forms and a physician is consulted. It is not always at an end with the formation of pus, as it may form and discharge again and again.

Fritsch mentions one case coming under his observation which continued to discharge pus in varying quantities for eight years. Of course, these cases are almost hopeless ones as far as radical cure is concerned, especially in the poor, who have neither time nor money to spend in the attempt to gain health as long as they are able to be about the house and attend to household affairs. What, then, would lead us to suspect this disease in the chronic form, which is the most difficult to diagnose? To partially recapitulate, the usual history is this: the patient has been confined within the last two to six months, but had not since that time regained her former health, often referring the circumstance to her nursing a child, when that takes place; is feeble, has poor appetite, nervous and gloomy, with a tendency to look at life on its dark side, an uneasy feeling and dragging sensation about the pelvis, with difficult defecation and possibly painful micturition. A digital examination will reveal a hardness and induration on both sides of the uterus, and generally in the cul-de-sac of Douglass as well, judging from my own experience, although most authors deny its presence in that locality in the majority of cases. It is sometimes found between the bladder and the uterus. If the examination is made with the disengaged hand pressing over the hypogastrium, the uterus will be found fixed and immovable by the deposition and adhesiveness of lymph. It may be confounded with pelvic peritonitis, hematocele and fibrous tumors. Like all other diseases which afflict humanity, absolute rest is one of the requisite factors in treatment, and especially so in this case. In the first stage, cooling drinks, with an aperient if there is constipation, should be resorted to, with opiates to control pain, which usually is not great. Poultices applied to the abdomen are indicated and are very grateful to the patient. Aconite or veratrum viride may be given, and during the height of the fever I have used antipyrin with apparent comfort to the patient and with the result of lowering the temperature. Blisters applied to the hypogastrium are highly beneficial, and recommended by the best authorities, when the acute stage has passed. I have also used suppositories of iodoform with satisfactory results, one to be placed in the vagina every eight hours. Iodide of potassium may also be given, or corrosive sublimate for the alterative effect when the disease merges into the chronic form. The hot water vaginal douche every few hours gives relief and tends to quiet inflammatory action. Should abscesses occur, free opening should be recommended when they can be reached, and the sac washed out with equal parts of tincture of iodine and water, as recommended by Barbour. I have only to report two cases coming under my own observation, and I have finished. Mrs. S., aged 26; mother of one child; had not menstruated for two months, when, on July 29, 1886, she aborted, followed in three or four days by pelvic pains, high fever and frequent pulse, great pain during micturition and defecation. She was seen by three different physicians up to September 1st, when she came into my hands. The first day I saw her she had a temperature of 102⅔, pulse 120, marked tympanitis, with a well-marked fluctuating abdominal tumor reaching to the umbilicus, which, previous to her coming under my care, had caused a diagnosis of pregnancy to be given. Digital examination revealed an induration of the entire pelvic contents, with a fixed uterus. Profuse sweating and chills led me to suspect pus formation, and I asked Dr. Thomas Lothrop to see the case with me. He did not believe her pregnant, but confirmed my diagnosis of pelvic cellulitis, and also thought there was present some pelvic peritonitis as well. The patient was now in an extremely critical condition, pulse ranging from 120 to 130. On September 13th, when I visited her I was told she had had a large movement from the bowels, containing pus and blood, but it had been thrown away. There was an amelioration of all the symptoms and a marked diminution in the size of the abdominal tumor. I directed should another such movement from the bowels occur that it be kept for my inspection, but through carelessness that also was disposed of. The abdominal tumor grew gradually smaller until it disappeared entirely, the active symptoms abated, the tympanitis subsided and the woman went on to a good recovery, although the uterus still has a fixed appearance. She has menstruated regularly since that time. April 5, 1887, called to Mrs. H., aged 27, mother of three children; found her aborting; used corrosive sublimate solution whenever necessary to make a vaginal examination. Three days after she was seized with chill followed by fever and delirium of rather a wild type. The pulse for several days was 130 per minute and temperature 104° F., with a constant bloody uterine discharge. Moderate tympanitis was also present. Excessive sweating and headache, coupled with delirium and diarrhœa, led me to believe her to be suffering from blood poisoning; washed out vagina with bichloride solution twice a day. I regarded death as imminent for three or four days, when she seemed to take a turn for the better. In a few days the right shoulder commenced swelling; was red and painful. The uterus was fixed with quite a tumor in Douglass' cul-de-sac. Dr. Thomas Lothrop also saw this case with me, and recommended blisters over the hypogastrium, hot water baths per vagina, and bichloride of mercury internally. The shoulder swelling went on to suppuration, was incised, discharged freely and is now healing slowly while another is forming over the tibia on the right leg. The uterus remains fixed in an indurated mass. Very little fever now and no pain except from abscess.

